# Exploring electronic cigarette portrayals: a content and thematic analysis of African online news coverage

**DOI:** 10.1186/s13011-023-00559-6

**Published:** 2023-08-29

**Authors:** Chimwemwe Ngoma, Yusuff Adebayo Adebisi

**Affiliations:** 1 Tobacco Harm Reduction Scholarship Programme, Knowledge•Action•Change, London, UK; 2 Department of Journalism and Media Studies, Malawi University of Business and Applied Sciences, Blantyre, Malawi; 3 Global Health Focus, Ibadan, Nigeria

**Keywords:** Portrayals, e-cigarettes, Electronic cigarettes, News, Content analysis, Thematic analysis, Vaping

## Abstract

**Background:**

Electronic cigarette use has surged internationally in recent years, with numerous countries noting an uptick in users. Despite this, the portrayal of e-cigarette usage in African news remains unclear.

**Methods:**

This research investigates the subject, employing a mixed-methodology approach. The study units were news articles on electronic cigarette use retrieved from AllAfrica, an online African news archive. A total of 38 online news and opinion articles published between June 2017 and June 2022 qualified the exclusion and inclusion criteria. A content analysis revealed prevalent codes and themes, while a thematic analysis explored the association between news sources and framing.

**Results:**

The results indicate that articles and arguments presenting e-cigarettes positively outnumber those with a negative slant. The health impacts of electronic cigarettes emerged as the most discussed topic, with health authorities frequently cited as news sources. However, these health authorities often lacked a unified stance on e-cigarette safety.

**Conclusion:**

The lack of consensus among health officials could have public health consequences, possibly resulting in the formulation of uninformed policies.

## Introduction

The use of electronic cigarettes, commonly referred to as vaping, has seen an exponential increase in acceptance and usage around the globe in recent years. Electronic cigarettes, devices that simulate the feeling of smoking by heating a liquid solution that usually contains nicotine, have become a novel lifestyle choice and a potential safer alternative to smoking [1] for many individuals. In 2018, the estimated number of electronic cigarette users was 58.1 million globally [2]. Indicating a significant shift in the tobacco market and a strong trend towards alternatives to traditional smoking. Specifically, within the African region, the vaping community consisted of approximately 4.1 million users [2], representing 7.1% of users globally. This number, while substantial, signifies the diverse regional trends in e-cigarette usage, influenced by factors such as local laws, cultural norms, and economic conditions. Electronic cigarettes have attracted attention due to their potential as a less harmful alternative to smoking, their rising popularity, and the regulatory challenges they pose [3].

While electronic cigarettes have been touted as potential tools for tobacco harm reduction, the scientific community remains divided over their safety, effectiveness for smoking cessation, and associated ethical implications [4]. There is considerable debate among public health professionals regarding these facets, underscoring the need for rigorous and comprehensive research to accurately assess the long-term health impact of e-cigarette use. Considering the skyrocketing popularity of electronic cigarettes, media attention on this topic has similarly soared [5]. The role of the media in shaping public perception and understanding of e-cigarettes, therefore, is substantial and consequential. This role is particularly pronounced in many parts of the world, especially in low and middle-income countries (LMIC), where the media is often the primary source of health information. The media stands as one of the most popular and cost-effective public health promotion tools available [6]. As such, it plays a pivotal role in disseminating critical health-related information to the public, shaping health behaviors [7], and influencing policymaking [8]. This underscores the importance of accurate and responsible media coverage of health issues, such as electronic cigarette use, to ensure that the public is well-informed and can make healthy choices based on reliable information.

Grasping the nature of media portrayals and their subsequent influence on audience perceptions is crucial, as research has pointed out inconsistent framing and tonal variations in news coverage of e-cigarettes [9]. This understanding is essential to ensure that individuals are making informed decisions based on accurate, balanced information rather than skewed media representations. Studies conducted in South Korea [5] and China [10] have indicated a substantial emphasis on the drawbacks of electronic cigarettes in media portrayals. Rather than discussing potential positive health outcomes, such as the use of e-cigarettes for smoking cessation, media narratives in these countries tend to accentuate the risks and potential harms associated with vaping. In contrast, research in England, Canada, and the United States of America (USA) [11] reveals an uptick in negative news stories concerning e-cigarettes and a corresponding increase in perceptions of vaping harms among youths. This suggests that media discourse can significantly influence public opinion, especially among younger demographics, and shape social norms and behaviors surrounding e-cigarette use. Interestingly, country-specific media arguments and narratives differ markedly. For instance, while media in the USA and United Kingdom (UK) stress the potential harm reduction benefits of e-cigarettes compared to traditional smoking, Korean media centers its discourse on the composition and potentially harmful ingredients of e-cigarettes [12]. This showcases the diversity of perspectives on e-cigarettes globally and underlines the role of media as a powerful amplifier of these varying viewpoints.

Notwithstanding the rising popularity of electronic cigarettes, there exists a dearth of knowledge regarding the framing of news concerning electronic cigarette use in Africa. Therefore, this study aimed to analyze news portrayals of electronic cigarette use in Africa with a focus on examining the key components of news coverage. Additionally, the study sought to gain a better understand the journalistic practices that shape news framing in the region, specifically on utilization of news sources. To achieve this, the study sought to address two questions: firstly, who is typically referred to as a news source, and secondly, what is the level of diversity of news sources.

## Methods

### Study design and article selection

This study utilized a mixed-methodology approach, combining both quantitative and qualitative methods, to provide a comprehensive exploration of news articles on electronic cigarette usage in African media. This was structured using an explanatory sequential design, a type of mixed methods design that starts with a quantitative data collection and analysis phase, followed by a qualitative phase to help explain or elaborate on the quantitative results.

The articles for this study, comprising both news reports and opinion pieces, were retrieved from the AllAfrica database. AllAfrica is a substantial online news archive that aggregates and disseminates around 600 reports online-only news and opinion articles a day from over 140 prominent African news organizations. This database was chosen because it offers a broad scope of media representation from across the African continent. The search strategy implemented for article selection involved the use of specific terms and phrases related to electronic cigarettes, such as “vaping,” “electronic nicotine delivery systems (ENDS),” and “electronic cigarettes.” This was done to ensure that the articles selected were directly relevant to the research topic.

The time frame for the selected articles was established as between June 2017 and June 2022. This period was chosen to provide a recent and relevant snapshot of the portrayal of e-cigarettes in African media. Additionally, these dates ensure that the research findings can contribute to the current discourse and provide a timely understanding of the topic. The keyword search yielded a total of 251 articles. The selection process involved a thorough screening of the articles obtained from the initial search. Duplicate articles, those not written in English, and pieces where e-cigarettes were not the primary focus were excluded. The restriction to English language articles was deemed appropriate given the predominant use of English as the language of instruction and official communication in many African countries. However, we acknowledge that our restriction to English language papers could potentially omit relevant articles in other languages, although the extent of this impact is expected to be minor. This resulted in a final sample of 38 articles that met the inclusion criteria. Each of these was then subjected to further analysis. This approach ensured a systematic and comprehensive selection of articles that were most relevant and representative for this study. The resulting sample provides a solid foundation for both the quantitative and qualitative phases of the research.

The study gathered news stories from numerous media outlets from various African nations, including those in the East Africa (Rwanda, Kenya), West Africa (Nigeria, Ghana), and Southern Africa (Malawi, Zimbabwe, South Africa, Namibia). Additionally, commentaries from the AllAfrica news organization, encompassing the entire continent of Africa, were included in the dataset. The highest number of articles, 14, were retrieved in 2020, followed by 8 in 2022, 6 in 2021, and 4 each in 2019 and 2018, with 2 articles in 2017.

### Quantitative analysis: content analysis

The first stage of the analysis leveraged the robust technique of quantitative content analysis, a popular method in media studies for systematically identifying, categorizing, and quantifying themes and frames in communication messages [13]. This approach allows for objective and systematic description of manifest content in the text. For the purpose of this research, a deductive approach was adopted to identify the themes and subthemes, with our understanding being guided and structured by previous research on the framing of e-cigarette usage in the media [3, 5, 10, 14]. This ensured that the coding scheme was theoretically informed and consistent with current discourse on e-cigarette framing. The main themes encompassed dominant frames (e.g., health-related, policy, economic, ethical and consumer interest frames), the tone (positive, negative, neutral) of the articles, and the main topics discussed (e.g., health risks or benefits, policy debates, economic considerations). In our analysis, the tone of each article was assessed based on a combination of factors, not simply a summation of health benefits and drawbacks. These factors included the context, overall argument, and the balance of positive and negative statements about e-cigarettes. An article could indeed have a negative tone overall even if it mentioned that e-cigarettes are a possible smoking cessation tool. The mention of a single benefit does not automatically classify an article as having a positive or neutral tone. To evaluate the tone, each article was read thoroughly by the research team and the overall impression of the article towards e-cigarettes was considered. In addition, to reduce bias and enhance the validity of our findings, a second independent researcher checked the tone assessments. Any disagreements were resolved through discussion until consensus was reached.

We categorized the main topic of each article based on the central narrative or discourse around e-cigarettes. While many articles discussed multiple facets of e-cigarettes, such as health effects, policy issues, and youth vaping, we classified them based on the aspect that was most emphasized or formed the crux of the article’s discussion. For instance, if an article mentioned policy measures like taxation but framed it within the context of preventing youth access to e-cigarettes, we classified it under ‘Youth Vaping’. Similarly, an article might mention health risks, but if the central argument was about the potential allure of e-cigarettes for young people, we again categorized it under ‘Youth Vaping’. In essence, our categorization considered not just the topics mentioned, but the primary angle from which these topics were approached in the article. We acknowledge that such classifications may have some degree of subjectivity, and we took careful steps to ensure consistency and reliability in our coding process.

NVIVO software, a qualitative data analysis computer software package produced by QSR International, was employed to facilitate the coding and thematic organization process. Each article was imported into NVIVO, and detailed codes were assigned based on the predefined themes and subthemes. These codes were applied to segments of text, such as sentences or paragraphs, which discussed the same idea. Coding was conducted independently by two experienced researchers (C.N and Y.A.A) to avoid any single researcher bias. Each coder underwent extensive training on the coding guide, which included clear definitions and examples for each code. The researchers held regular meetings to discuss and resolve any discrepancies, enhancing the intercoder reliability. Additionally, a third independent coder was available to settle any unresolved coding disagreements, ensuring the robustness and consistency of coding.

Following the content analysis, the coded data was exported to Microsoft Excel. This step facilitated the quantification of coded themes, allowing us to identify frequencies and draw comparisons between various categories. Excel was also employed for conceptual mapping, a graphical tool used to visualize relationships and trends within our data, facilitating a better understanding of the patterns and insights gleaned from the content analysis.

### Qualitative analysis: thematic analysis

The second stage of our analysis was grounded in thematic analysis, a widely used qualitative research method. This method, involves identifying, analyzing, and interpreting patterns of meaning (‘themes’) within qualitative data, providing a rich and detailed, yet complex account of the data [15]. The thematic analysis for this study was primarily inductive, often referred to as a ‘bottom-up’ approach, where the themes identified are deeply rooted in the data itself. The researchers thoroughly engaged with the data by repeatedly reading and reviewing the articles, noting initial ideas. This process fostered an environment where themes could be identified through an iterative process of analysis, which allowed for an understanding of the data in its own right and in the context of the researchers’ existing knowledge and perspectives. This approach acknowledges that while the themes were not predetermined by the researchers, their interpretation was inevitably shaped by their existing knowledge and understanding. This balance between data-driven analysis and researcher interpretation is a crucial aspect of inductive thematic analysis.

Our analytical focus centered on journalistic practices, particularly regarding the selection and use of news sources in the coverage of electronic cigarettes. The researchers sought to answer two questions: Who is typically referred to as a news source in these articles? And what is the diversity level among the sources in news on e-cigarettes? This focus on source diversity was crucial, as it sheds light on the range of perspectives and voices being included in the discourse, which can significantly impact how e-cigarette use is framed. After familiarization with the data, the researchers systematically generated initial codes across the entire data set, capturing interesting features pertinent to our research questions. These codes were then collated into potential themes, gathering all data relevant to each potential theme. The researchers then reviewed these themes, refining them further. This stage often required some back-and-forth between coding and theme generation, ensuring a good fit between the coded extracts and the defined themes.

This iterative process resulted in a robust thematic framework that captured the complexities of news source selection and usage. The themes were named and defined, and compelling extract examples were chosen to demonstrate the nature and scope of each theme. Finally, a detailed analysis was produced, relating our findings back to the research questions and existing literature, providing a clear understanding of the journalistic practices around e-cigarette coverage in African media.

## Results

The study reveals that half of the articles (n = 19, 50%) have a favorable tone towards e-cigarettes. Furthermore, the majority of the articles were framed as a health issue (n = 22, 58%). In terms of diversity of sources, the analysis showed that 31 articles (81.6%) used two or more sources, while 7 articles (18.4%) used only one source. Additionally, the most commonly used news sources in articles on e-cigarettes are public health agencies, seconded by retailers/manufacturers. Table 1 provides an overview of the frequency of different frames within the analyzed articles. It breaks down themes and sub-themes, indicating the main theme count and mentions count.

**Table 1:** Frequency of frames

Themes and sub-themes		Main theme count	Mentions count	Percentage
**Health impacts of electronic cigarettes**				
Benefits of e-cigarettes		28		62.22%
	Smoking cessation tool		8	-
	Reduction in smoking-related diseases		8	-
	Safer as compared to smoking		24	-
	Not linked to chronic illnesses		3	-
Drawbacks of e-cigarettes		17		37.78%
	Not an effective smoking cessation tool		4	-
	E-cigarettes are a gateway to smoking		2	-
	E-cigarettes are harmful		9	-
	E-cigarettes are addictive		8	-
	Second hand aerosols are harmful		2	-
	Potentially harmful		4	-
	Youths and children taking up e-cigarettes		7	-
**Story tone typically used**				
Neutral		8		21.05%
In favor of e-cigarettes		19		50%
In opposition to e-cigarettes		11		28.95%
**Main topic**				
Policy issue		13		34.2%
	Products taxation		8	-
	Product regulation		21	-
	Product ban		11	-
Health issue		22		58%
Youth vaping		3		7.8%
**News source**				
Government officials/ lawmakers			8	9.09%
Public Health Agencies			19	21.59%
Anti-vaping advocates and CSOs			4	4.55%
Vaping advocates and CSOs			11	12.50%
Average citizen/consumer			3	3.41%
Industry – retailers and manufacturers			14	15.91%
Health experts – doctors/ nurses			12	13.64%
Academia			11	12.50%
Journals on health			6	6.82%

### Dominant frames

The frame that electronic cigarettes are safer as compared to smoking was the most frequent, it appeared in 24 articles, followed by the frame which portrays electronic cigarette use as a health issue where the benefits and drawbacks of electronic cigarettes were discussed and appeared in 22 articles. Below are some of the excerpts that referred electronic cigarettes to as a safer alternative to smoking.

“In 2015 Public Health England (PHE) reported that “an expert review of the latest evidence concludes that e-cigarettes are around 95% safer than smoked tobacco and they can help smokers to quit” *(Africa Check, 2022).*

“Scientific research led by independent experts and professors have shown through the years the great potential of less harmfulness of e-cigarettes” *(All Africa, 2022).*

The second dominant frame was on the regulation of electronic cigarettes, and appeared in 21 articles. The articles covered the debate on regulation in which other articles advocated for sensible regulation of the product whilst other articles took a prohibitionist approach and demanded bans or stricter regulation:

“Preventing access to these products through bad policies robs people of their right to health. Policy should encompass issues to do with access to credible information/science and making Tobacco Harm Reduction products available” (*AllAfrica, 2020).*

“… e-cigarette products actually require stricter or a step up in regulation to match the sophistication and diversity of the products and ensure that other innovations the industry might come up with in the future are covered” (*Daily Maverick, 2020).*

### Tone typically used

The tone was the stance that had been used to describe the whole article. Classified as favorable towards electronic cigarettes, unfavorable towards electronic cigarettes and neutral, 19 articles portrayed electronic cigarettes in a favorable way where the benefits of electronic cigarette use were presented as a safer alternative to smoking and as effective smoking cessation aids.

On the other hand, 11 articles were in opposition to electronic cigarette use, and negative attributes of electronic cigarettes were underscored including that electronic cigarettes are a gateway to smoking for non-smokers and children, equally harmful as smoking and that electronic cigarettes are addictive and capable of causing dependency.

Only eight articles appeared to have a balanced tone where both positive and negative attributes of electronic cigarettes were discussed. In this way, the authors of these articles gave the audience the opportunity to critically assess the available evidence and come to their own conclusions regarding the potential effects of electronic cigarettes. This approach indicates a commitment to scientific inquiry and evidence-based analysis, which are critical to facilitating informed decision-making on the part of both individuals and policymakers. Additionally, it underscores the importance of avoiding dogmatic or one-sided narratives in favor of a more balanced and objective approach that considers all available evidence.

### Benefits and drawbacks of electronic cigarettes

Overall, the benefits of electronic cigarette use appeared more times than drawbacks and appeared in 28 articles and 17 articles respectively. For example, the frame that e-cigarettes are safer as compared to smoking appeared in 24 articles as compared to the frame that they are harmful which appeared nine times. Furthermore, the argument that electronic cigarettes are an effective smoking cessation tool (eight articles) outnumbered the argument that they are not an effective smoking cessation tool (four articles).

Some of the arguments indicating that electronic cigarettes are effective smoking cessation tools include:

“Two of the reviews found that vaping was more effective for quitting smoking than other methods, including nicotine replacements like patches or chewing gum. A 2021 report by PHE found that across multiple reviews, vaping was more or equally as effective as other tools” *(Africa Check, 2022).*

“Harm reduction is an evidence-based approach to tobacco control, which, along with other proven tobacco control interventions, can simultaneously prevent youth from starting to smoke and help current smokers stop, saving many lives more quickly than would otherwise be possible” *(All Africa, 2020).*

On drawbacks, the most common appearing frame is that electronic cigarettes are harmful and it appeared in nine articles followed by the frame that electronic cigarettes are addictive which appeared in eight articles.

Some articles argued that electronic cigarette use causes a number of non-communicable diseases including various cancers, cardiovascular diseases and pulmonary diseases:

“Electronic cigarettes and heated tobacco products are not helping to fight cancer, the World Health Organisation (WHO) said on Friday, urging smokers and governments not to trust claims from cigarette manufacturers about their latest products” *(The Namibian, 2019).*

“Both cigarette and electronic cigarette (e-cigarette) use, damages the respiratory system, potentially increasing the risk of experiencing Covid-19-related symptoms, a positive diagnosis and exacerbated health outcomes” *(Nation, 2020).*

Further to that, some articles argued that electronic cigarettes can hook citizens to nicotine addiction and that they are prone to explosions which can cause injury to users:

“… the introduction of new products beyond e-cigarettes has led to addictions which are negatively affecting the health of citizens” *(Capital Business, 2022).*

“there are still many strong criticisms against tobacco harm reduction, notably from the World Health Organization. Some of the disapprovals include: the continued promotion of an addictive substance which is nicotine even though it is not carcinogenic, an unproven long-term safety, an unclear efficiency for smoking cessation and reduction and last but not least, a gateway to attract the youth” *(All Africa, 2020).*

### Main topic

In relation to the overall story topic, the articles were categorized into three; health issue, policy issue and youth vaping issue. Articles on health issue as the main topic appeared in 22 articles. In these articles, the benefits and drawbacks of electronic cigarettes were the center of discussion.

The second dominant angle was policy issues. Appearing in 13 articles, the discourse was focused on the regulation of electronic cigarettes which included tax measures, accessibility and affordability issues and whether the prohibition of the product would be ideal.

“We see a barrier ahead. We ask Parliament not to do what they did last time (2021) by lowering proposed taxes. Parliament should even increase the amount and come up with more stringent measures. It should not try to please the tobacco industry. If it wants to make any changes, it must consult the Ministry of Health” *(Capital FM, 2022).*

“If the proposals will be passed by the August house, the e-cigarettes, also known as vaporizers or vapes will cost more so as to ensure they are not easily available” *(Capital FM, 2022).*

The youth vaping issue came third and it was the main topic in 3 articles only which they were argued to be more appealing to the youth. Governments were further implored to protect the health and well-being of youths by putting in place measures that discourage them from taking up electronic cigarette use:

“In order to prevent these habits and make the liquid nicotine used in these devices less accessible to users including to school children and the youth, I propose to change the taxation regime” *(Capital Business, 2022).*

“In fact, the WHO affirms that its main concern with these nicotine alternatives to cigarettes are that they may be too risky because they could appeal to the youth” *(All Africa, 2022).*

### News sources

The study found that among the 38 articles in our corpus, public health agencies at national, regional, and global levels were the most commonly referenced sources, appearing in 19 articles. Retailers and manufacturers were cited in 14 articles, making them the second most common source. Health officials, such as doctors and nurses, were cited in 12 articles. When we analyzed the tone associated with each source, we found some patterns. Retailers and manufacturers generally expressed a positive view of electronic cigarettes in 93% (13 out of 14) of the articles where they were cited. They commonly portrayed e-cigarettes as safer alternatives to traditional tobacco products. On the other hand, public health officials offered more nuanced views. While they were the most frequently cited source, the tone of their commentary varied. They advocated for the use of e-cigarettes as a safer alternative to traditional smoking and an effective cessation aid in about 37% (7 out of 19) of the articles where they were mentioned. However, in approximately 63% (12 out of 19) of these articles, they criticized e-cigarettes, highlighting them as potentially harmful, addictive, and a potential gateway to smoking for non-smokers and the younger generation. These patterns illustrate a clear difference in perspectives between industry representatives and public health officials on the issue of e-cigarettes.

### Diversity of news sources

The study revealed that out of a sample of 38 articles, 31 articles had two or more sources appearing, whereas a minority of seven articles relied solely on a single source throughout the article. It is noteworthy, however, that the presence of multiple sources did not necessarily indicate a balanced perspective and tone in the articles. In some instances, all sources cited in the article were found to adopt a negative view of electronic cigarettes, while in other cases, all sources exclusively emphasized the positive aspects of electronic cigarette use.

## Discussion

This study found that positively framed articles and arguments about e-cigarettes in Africa outnumbered negatively framed articles and arguments. These findings align with similar patterns observed in Indonesian news media [14] and in news media in USA, UK, and Korea [12]. Furthermore, the three studies share similarities in terms of the most discussed topic, which centered around the health impacts of electronic cigarettes. This could be due to the increasing amount of scientific knowledge about the health effects of electronic cigarettes in recent years.

### A growing body of science and evidence

This research study reports that a majority of the news stories (n = 19) analyzed focused on health issues related to electronic cigarettes, discussing both their benefits and drawbacks. Public health agencies have conducted extensive research on the safety of electronic cigarettes since their introduction to the market. Notably, Public Health England’s landmark 2015 study concluded that using electronic cigarettes is 95% safer than smoking [16]. This finding has been subsequently endorsed by other prominent public health bodies, including the Royal College Physicians, the International Agency for Research on Cancer, the British Medical Association, and Cochrane Review.

Additionally, this study reveals that half of the articles (n = 19, 50%) exhibit a favorable tone towards electronic cigarettes. News sources in favor of electronic cigarettes frequently invoked Public Health England’s findings in their arguments, which emerged as the most commonly employed frame under the health impact theme. However, some health officials and scientists remain skeptical regarding the safety of electronic cigarettes. Other scholars also argue that more scientific evidence is needed on the relative safety of electronic cigarettes and cautions the media to be careful not to disseminate news that is not based on evidence [10].

### Lack of consensus among health officials

This study observed an apparent lack of unison in argument about electronic cigarettes between health authorities that were used as news sources. While other public health agencies, researchers, and health officials advocated the use of electronic cigarettes as a much safer option and a means of smoking cessation, other health agencies at the national, regional, and global levels flatly dismissed the argument. For instance, the argument that electronic cigarettes are safer as compared to smoking appeared in 24 articles and the argument that electronic cigarettes are equally harmful appeared in 9 articles.

Public Health England is an example of a reputable public health agency that have conducted independent research to scientifically validate the effectiveness of electronic cigarettes and has since taken a more welcoming stance toward e-cigarette use as a tool for smoking cessation. The World Health Organization (WHO), on the other hand, has a different perspective and has voiced its concern over the lack of regulation and potential health risks associated with e-cigarettes [17].

The ongoing debate on whether or not electronic cigarettes are a healthier option not only indicate a lack of scientific consensus among health leaders at the national, regional, and global levels but also reflects differing priorities in public health strategies. The divergent perspectives surrounding this matter suggest that either the proponents or detractors of electronic cigarettes are disseminating potentially inaccurate information, thereby posing consequential risks to public health [5]. Moreover, these conflicting viewpoints highlight a broader issue of balancing health risks and benefits for different segments of the population. A lack of consensus on whom to prioritize and the appropriate trade-offs to make between the health of various population segments further complicates the issue [18, 19]. Particularly in the African context, socio-economic factors and distinct health realities add an additional layer of complexity to these discussions.

On the other hand, critics argue that some of the studies claiming electronic cigarettes are a healthier option are funded by tobacco companies and thus question the findings [20]. However, it may be contended that studies executed with transparent methodologies and with results verifiable through replication warrant consideration as valid.

### Use of news sources and main topic used

News sources play a significant role in shaping the content of the story by providing frames of interpretation to news writers [21]. A knowledgeable reader can easily and accurately predict the content of a story by looking at news sources [5].

This study found that public health institutions (n = 19) were the most involved type of news source, seconded by retailers and manufacturers (n = 14) who are likely to have a favorable view towards the product which they produce, electronic cigarettes. This probably explains why the health issue was the most salient theme and most stories were framed with a positive tone toward electronic cigarettes.

On the contrary, previous studies on content analysis on electronic cigarettes in Chinese newspapers [10] and South Korean news media [5], found that electronic cigarette use was viewed more as a political issue than a health issue, and the main news sources were government officials and legislators. These news sources are likely to discuss and focus on regulatory issues surrounding the use of electronic cigarettes than clinical and scientific issues that the products raise.

While electronic cigarette use is an issue for both policymakers and healthcare professionals as key stakeholders who champion the regulatory issues and safety of the products, news writers often ignore or fail to capture the perspectives of electronic cigarette users, who are also key stakeholders in this discourse. The consumers have first-hand experience with the product and their insights could prove invaluable in shaping e-cigarette regulations. However, this study found that only three out of the 38 articles used consumers as a news source, indicating a lack of representation of their perspective. It is crucial for news coverage of electronic cigarettes to include a variety of perspectives because the status quo suggests that people’s perceptions of electronic cigarettes may be shaped more by the perspectives of social-political elites than the voices of users who have firsthand knowledge of using electronic cigarettes [9].

The study indicates that journalistic practices employed by news writers in selecting news sources have significant implications on the salient frames and perspectives of a news story. From this, the following three conclusions can be drawn; if public health officials are used as key sources, the story is likely to be presented as a health issue. Secondly, when government officials and legislators are used as the primary source, policy issues are likely to stand out. Finally, when retailers and manufacturers are used as the primary source, the story is likely to have a positive tone.

### Study limitations

Our study has several limitations that should be noted. Firstly, the restriction to English language articles may potentially exclude relevant data from non-English African news sources. This was necessary to maintain consistency and feasibility of our analysis but may limit the diversity of our dataset. Secondly, we acknowledge that our categorization of the main topic of each article may have some degree of subjectivity. While we made rigorous efforts to ensure consistent and reliable coding, the nuanced nature of these categorizations means they may not fully capture the multifaceted discourses around electronic cigarettes. Additionally, this study focused on content from the AllAfrica database, and the findings may not be generalized to all African media outlets, especially the North African media since we did not review any article from the region.

Lastly, although our mixed-methods design offered a robust understanding of the portrayal of e-cigarettes in the media, the quantitative nature of content analysis may not fully capture the complexity and subtlety of media messages. For instance, the determination of an article’s tone was based on the researchers’ assessment, which may vary among different individuals. Despite this, the use of multiple researchers for independent assessments helped to minimize bias.

### Future research directions

Given these limitations, future research could extend this work in several ways. Firstly, a similar study could be conducted using non-English African media sources to ascertain if findings differ significantly from our results. It would also be valuable to explore media coverage of electronic cigarettes in specific African countries, providing a more localized understanding of this issue. Secondly, to mitigate the potential subjectivity in determining the main topics of articles, more sophisticated text analysis techniques, such as machine learning and natural language processing, could be employed. These methods could help to further objectify the categorization process and uncover subtle, complex patterns in the data. Lastly, future research could delve deeper into the portrayal of e-cigarettes in different types of media, such as social media or blogs, to gain a more comprehensive understanding of the discourse around electronic cigarettes. This could provide valuable insights into public perceptions and attitudes towards e-cigarettes, informing health communication strategies and policy decisions.

## Conclusion

The study revealed an emphasis on the health impacts of electronic cigarettes as the most widely discussed topic. The utilization of empirical evidence and scientific discourse in the articles is a positive indication. Such a trend aids in shaping public perception and simultaneously provides valuable insights for the formulation of policies that uphold public health. However, the absence of consensus among health authorities on the health effects of e-cigarettes poses a threat to public health. It can create confusion among policymakers, nicotine users, and the public, resulting in negative public health outcomes. Therefore, it is important that scientists, researchers, and public health officials, regardless of their affiliations, promote credible science and evidence. The accuracy and completeness of media coverage of electronic cigarette use also depend on the rigorous and cautious assessment of news sources by news writers. To do this, news writers must consider both the credibility and diversity of news sources to capture multiple perspectives.

## Data Availability

The datasets used and/or analyzed during the current study are available from the corresponding author on reasonable request.

## Abbreviations

LMIC: Low and Middle-Income Countries
PHE: Public Health England
UK: United Kingdom
USA: United States of America
WHO: World Health Organisation
